# Age-specific risk factors of depression among the oldest-old - evidence from the multicenter AgeCoDe-AgeQualiDe study

**DOI:** 10.3389/fpsyt.2024.1367225

**Published:** 2024-06-11

**Authors:** Melanie Luppa, Alexander Pabst, Margrit Löbner, Tina Mallon, Christian Brettschneider, André Hajek, Kathrin Heser, Luca Kleineidam, Siegfried Weyerer, Jochen Werle, Michael Pentzek, Dagmar Weeg, Edelgard Mösch, Birgitt Wiese, Anke Oey, Michael Wagner, Wolfgang Maier, Martin Scherer, Hans-Helmut König, Steffi G. Riedel-Heller

**Affiliations:** ^1^ Institute of Social Medicine, Occupational Health and Public Health, Leipzig University, Leipzig, Germany; ^2^ Department of Primary Medical Care, Center for Psychosocial Medicine, University Medical Center Hamburg, Eppendorf, Germany; ^3^ Department of Health Economics and Health Services Research, Hamburg Center for Health Economics, University Medical Center Hamburg, Eppendorf, Germany; ^4^ Department of Neurodegenerative Diseases and Geriatric Psychiatry, University Hospital, Bonn, Germany; ^5^ German Center for Neurodegenerative Diseases (DZNE), Bonn, Germany; ^6^ Central Institute of Mental Health, Medical Faculty, Mannheim, Germany; ^7^ Institute of Family Practice (ifam), Medical Faculty, University of Duisburg, Essen, Germany; ^8^ Department of Psychiatry, Technical University of Munich, Munich, Germany; ^9^ Institute of General Practice, Hannover Medical School, Hannover, Germany

**Keywords:** incidence, predictors, risk factors, competing risk, depression, depressive symptoms, old age, late life

## Abstract

**Purpose:**

The present study aimed to investigate age-group-specific incidence rates and risk factors for depressive symptoms in the highest age groups.

**Methods:**

Data were derived from a prospective multicenter cohort study conducted in primary care – the AgeCoDe/AgeQualiDe study. In total, 2,436 patients 75 years and older were followed from baseline to ninth follow-up. To assess depressive symptoms, the short version of the Geriatric Depression Scale (GDS-15, cutoff score 6) was used. Age-specific competing risk regressions were performed to analyze risk factors for incident depressive symptoms in different age groups (75 to 79, 80 to 84, 85+ years), taking into account the accumulated mortality.

**Results:**

The age-specific incidence rate of depression was 33 (95% CI 29-38), 46 (95% CI 40-52) and 63 (95% CI 45-87) per 1,000 person years for the initial age groups 75 to 79, 80 to 84 and 85+ years, respectively. In competing risk regression models, female sex, mobility as well as vision impairment, and subjective cognitive decline (SCD) were found to be risk factors for incident depression for age group 75 to 79, female sex, single/separated marital status, mobility as well as hearing impairment, and SCD for age group 80 to 84, and mobility impairment for age group 85+.

**Conclusion:**

Depressive symptoms in latest life are common and the incidence increases with increasing age. Modifiable and differing risk factors across the highest age groups open up the possibility of specifically tailored prevention concepts.

## Highlights

- Adjustment for competing mortality in determining factors of late life depression.
- Different age-group specific risk factors in in late life depression.
- Significant differences in age-group specific incidence rates in late life depression.

## 1 Introduction

The demographic change and the pandemic situation create an increasing public awareness of the importance of mental health in the highest age groups. In particular, the frequency of the appearance of depressive symptoms in the oldest age groups and their adverse link to quality of life (1), physical comorbidity (2), and health care utilization (3) are a focus of high interest in research literature. Depressive symptoms are known to be common in old age (4); they lead to a reduced quality of life (5) and an increased health care utilization (3), and are more frequently accompanied by somatic complaints than in earlier adulthood (6).

However, information on the occurrence and risk factors for depressive symptoms in the oldest age groups is rare, since longitudinal studies require elaborate processing. In systematic reviews of the international literature on risk factors for depression in old age, only a few studies in the oldest age groups were reported (7–9). A current systematic summary of Maier et al. (4) reported only five studies including individuals from the middle-old age to the oldest-old age [75+: 10–14], and only one study conducted in a sample of oldest-old individuals [85+: 15]. Several risk factors for incident depressive symptoms were summarized (4), such as increased age, female sex, cognitive decline, functional impairment, and comorbid diseases.

Furthermore, only a few studies that analyze the incidence of late life depression adjusted for accumulated mortality over time (4), although mortality is naturally a very common competing event in old age, and indicated that female sex, marital status, subjective cognitive decline, and mobility impairment are strong risk factors for depressive symptoms. A recently published study investigated the incidence and risk factors of depressive symptoms in the entire oldest age population using data from the AgeCoDe/AgeQualiDe study such as the present study adjusting also for the competing event mortality (16), however, without considering different age groups.

Therefore, the present study aims to

(1) examine age-specific incidence rates of depressive symptoms across different age groups in late life, and
(2) analyze age-specific risk factors for incident depressive symptoms for these age groups using multivariate regression models adjusting for the competing event of mortality.

## 2 Methods

### 2.1 Study design

Data of the present study were derived from the German study on Ageing, Cognition, and Dementia in Primary Care Patients (AgeCoDe study), a prospective, longitudinal multicenter cohort study, and from the Study on Needs, Health service use, Costs, and Health-Related Quality of Life (AgeQualiDe study), the extension/continuation of the AgeCoDe study).

At baseline of the AgeCoDe study, 138 general practitioners (GPs) in six German cities (Bonn, Düsseldorf, Hamburg, Leipzig, Mannheim, and Munich) recruited study participants. Inclusion criteria were ≥ 75 years of age, no dementia diagnosis, and at least one GP contact within the previous 12 months. Exclusion criteria were German language insufficiency, consultation with GP at home only, residence in a nursing home, severe illness that GP would consider fatal within 3 months, deaf or blind, inability to provide informed consent, and being an irregular patient of participating practice.

**Figure 1:**
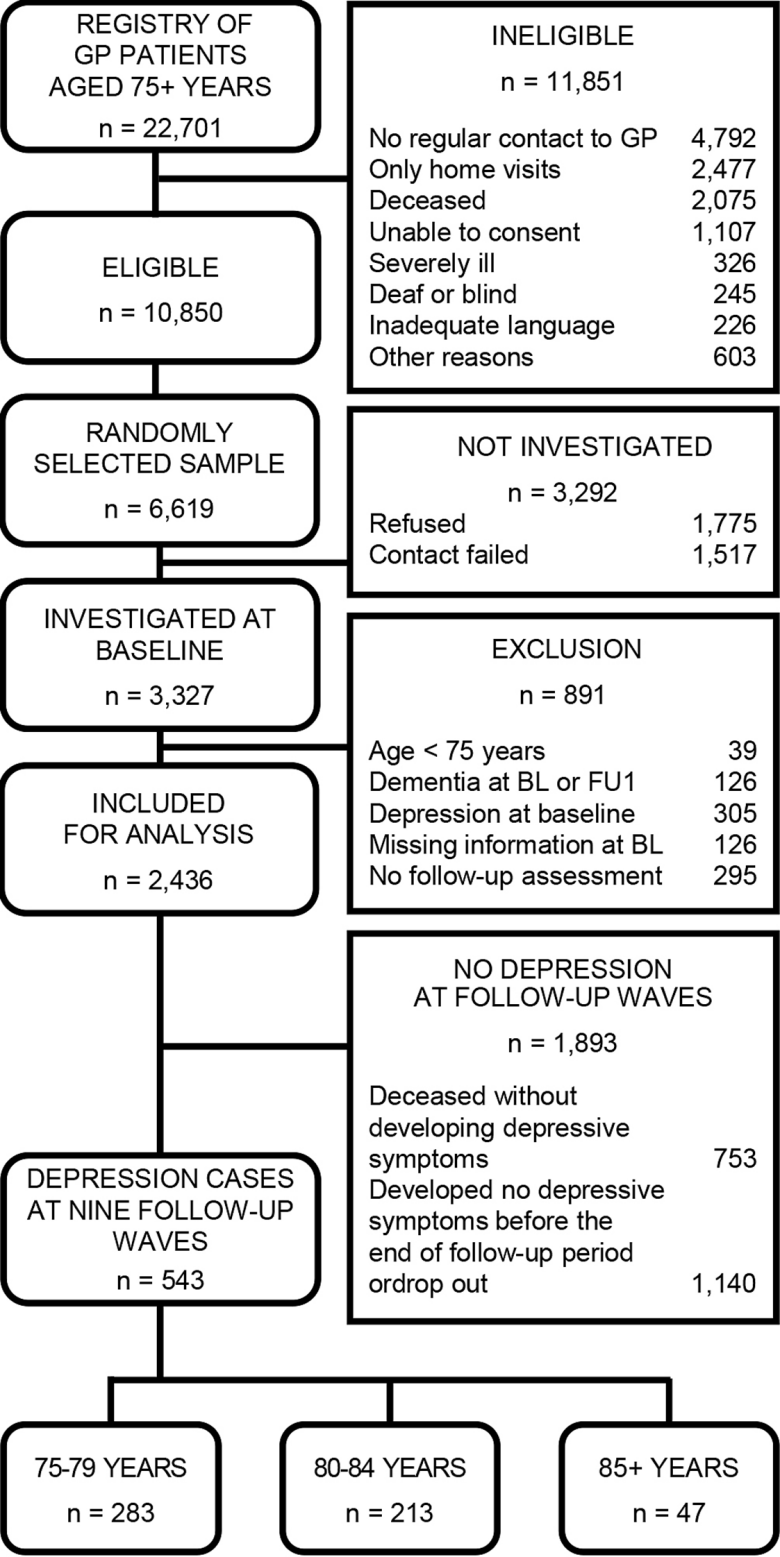
Flowchart of the sample selection process.

The AgeCoDe/AgeQualiDe cohort consists of n = 3,327 GP patients. In total, n = 891 patients had to be excluded: 39 (1.2%) individuals with an age under 75 years, 126 (3.8%) with a diagnosis of dementia at baseline or follow-up 1, 126 (3.8%) with missing information at baseline; 295 (8.9%) without assessment at follow-up 1. Additionally, 305 (9.2%) participants were above the cutoff point of 6 points in the GDS at baseline. Finally, 2,436 (73.2%) individuals were included in the analytical sample. More information on the sampling frame, eligible subjects, and respondents is presented in **Figure 1**.

#### 2.1.1 Ethics

All participants gave their informed written consent. The study protocols of both the AgeCoDe and the AgeQualiDe study have been approved by the ethics committees of all participating study centers and comply with the ethical standards of the Declaration of Helsinki (for details, see **Supplementary File 1**).

### 2.2 Data collection and assessment procedures

Data collection was carried out between January 2003 and November 2016 for baseline and nine follow-ups. After baseline assessment, study participants were followed every 1.5 years for follow-ups 1 to 7, and every 10 months for follow-ups 8 and 9. Further study details have been described elsewhere (17).

At the participants’ homes structured clinical interviews were conducted by trained physicians and psychologists.

A standardized interview included information on sociodemographic characteristics such as age, sex, marital status, living situation, and level of education using the Comparative Analysis of Social Mobility in Industrial Nations (CASMIN) classification (18).

Cognitive function was assessed using the Mini Mental State Examination (MMSE) (19), a short screening assessment to measure global cognitive function including items on orientation registration, attention and calculation (19 items, total score range 0-30). Subjective cognitive decline (SCD) was assessed before the assessment of cognitive status with the following question: ‘Do you feel as if your memory is getting worse (yes/no); if so, ‘Does this concern you?’ (yes/no). According to that, the participants were divided into three groups: no SCD, SCD without related worries; SCD with related concerns.

Impairment in complex instrumental activities of daily living (IADL) was assessed using the Lawton and Brody IADL scale (20), which contains in total eight items. Only information on the five items, which were assessed for men and women (ability to use the telephone, handle routine finances, use public transport, shop daily supplies, and be able to handle their own medication), was included. Impairment in at least one category led to a classification as ‘impaired’. The IADL scale shows an excellent inter-rater reliability (r=.99), and a good test-retest reliability (r. = 93) (21).

Furthermore, self-rated impairment of vision, hearing, and mobility was assessed by a standardized interview with a four-point Likert scale (none; mild; severe; profound). Due to the small number of cases recorded in the highest categories (e.g. 0/4/13 patients reporting profound impairment of hearing/vision/mobility), it was differentiated only between two categories (impaired=mild/severe/profound impairment *vs*. not impaired=no impairment).

Substance use was assessed for nicotine (never/former/current smoking) and alcohol (non-risky drinking/risky drinking: >12/>24 grams of pure alcohol per day (gpd) for women/men (22).

For each participant, the GP responded to a questionnaire with 15 chronic conditions (yes/no) (eg, diabetes mellitus, cardiac diseases, epilepsy, Parkinson’s disease, stroke, renal failure). Somatic comorbidity was defined as no comorbidity/1-4 diagnoses/5+ diagnoses according to a similar analysis for reasons of comparability (16).

The genotyping of apolipoprotein E (apoE) was performed according to standard procedures (23). In the analyzes, subjects were divided by ApoE status into those with or without at least one *ε*4 allele.

#### 2.2.1 Measurement of depressive symptoms

The 15-item version of the Geriatric Depression Scale (GDS-15) (24) was used to diagnose depressive symptoms. The GDS has a range from 0 to 15 points, has a simplified yes/no response format, and excludes questions for somatic symptoms. Therefore, it is especially suitable for the older and oldest age population. Friedman et al. (25) showed satisfying psychometric properties for the questionnaire. For the German version, a cutoff score of 6 yielded the best sensitivity (84%) and specificity (88.9%) to indicate clinically significant depressive symptoms (22). The average discriminatory power (.49), the average questionnaire difficulty (P = 43), the low interitem correlation (r = .19), and the high internal consistency (Cronbach alpha = .91) indicate that the German version of the GDS has good psychometric properties (26).

### 2.3 Statistical analyzes

Statistical analyzes were performed with Stata 15.1 MP (StataCorp LP, College Station, TX, USA). The level of statistical significance was established at p<.05 (two-tailed) for all analyzes.

Incidence rates of depressive symptoms were estimated for each age group as the number of cases that crossed the defined GDS-15 cutoff point of 6 points at follow-up divided by person years at risk. Since the aim of the present analysis is to examine age-specific risk factors for depression, the analytical sample was divided into three age groups: 75-79, 80-84 and 85+ years. For participants with incident depressive symptoms at follow-up, person years at risk were calculated as the time between the baseline visit and the follow-up interview with the first onset of depressive symptoms above the defined cutoff. For those who did not develop depressive symptoms during the study course, person-years at risk were calculated as the time between baseline visit and the last follow-up at which the participant could be attended.

Group differences were analyzed using Kruskal-Wallis equality of population tests for nonparametric quantitative measures and Pearson’s chi square tests for frequency comparison.

The risk factors for incident depressive symptoms were assessed using competing risk regression models (models I to III for age groups 75 to 79, 80 to 84, and 85+ years). The decision to compare risk regressions was made because it provides a useful alternative to Cox regression for survival data in the presence of competing events such as mortality (27), and should be preferred as an appropriate model for prediction research (28). Competing risk models are considered a useful alternative to the commonly used Cox models because death in our old age sample naturally accumulated over the course of the study, and this competing event may prevent the occurrence of future depression.

The selection of risk factors for the analysis was hypothesis-driven based on the findings and reported shortcomings of the studies in a current review of risk factors for depression in the elderly (4). Thus, the regression models included baseline information on age, sex, marital status, living situation, educational level, MMSE score, SCD, IADL impairment, impairment in vision, hearing and mobility, smoking, alcohol consumption, somatic comorbidity, APOE ϵ4 as independent variables. Adjusted subdistribution hazard ratios (sHR) with 95% confidence intervals (95% CI) were reported for depression as the event of interest, since mortality can also occur over time (28).

## 3 Results

### 3.1 Sample

In total, the study sample consisted of 2,436 individuals (73.2%; **Figure 1**). The mean age was 79.5 years (SD=3.5 years; age range 75-96 years). The three age groups 75 to 79 years, 80 to 84 years, and 85+ years comprised n = 1,339, n = 901 and n = 196 participants at baseline. The baseline characteristics of the three subsamples are shown in **Table 1**. Significant differences were found in sex, marital status, living situation, educational level, cognitive status, IADL, and smoking.

**Table 1:** Age-specific baseline characteristics of the study sample of GP patients for the total sample, and the age groups of 75 to 79 years, 80 to 84 years and 85+ years (N=2,436).

Characteristics at baseline	Total sample n=2,436	75-79 years n=1,339	80-84 years n=901	85+ years n=196	Test statistics	p value
Sex, n (%)						
Female	1,551 (63.7)	814 (60.8)	606 (67.3)	131 (66.8)	χ^2 ^= 10.7	**<.01**
Male	885 (36.3)	525 (39.2)	295 (32.7)	65 (33.2)		
Marital status, n (%)						
Single/divorced	278 (11.4)	143 (10.7)	108 (12.0)	27 (13.8)	χ^2 ^= 95.7	**<.001**
Married	1,070 (43.9)	697 (52.0)	329 (36.5)	44 (22.5)		
Widowed	1,088 (44.7)	499 (37.3)	464 (51.5)	125 (63.8)		
Living situation, n (%)						
Not alone	1,213 (49.8)	751 (56.1)	396 (44.0)	66 (33.7)	χ^2 ^= 53.9	**<.001**
Alone	1,223 (50.2)	588 (43.9)	505 (56.0)	130 (66.3)		
Level of education^1^, n (%)						
Low	1,476 (60.6)	831 (62.1)	541 (60.1)	104 (53.1)	χ^2 ^= 18.4	**<.01**
Middle	672 (27.6)	346 (25.8)	248 (27.5)	78 (39.8		
High	288 (11.8)	162 (12.1)	112 (12.4)	14 (7.14)		
MMSE score, mean (s.d.)						
	27.7 (1.7)	27.8 (1.7)	27.5 (1.8)	27.1 (1.8)	χ^2 ^= 36.8	**<.001**
SCD, n (%)						
No	1,046 (42.9)	582 (43.5)	390 (43.3)	74 (37.8)	χ^2 ^= 3.1	.537
Yes, but without related worries	1,046 (42.9)	564 (42.1)	387 (43.0)	95 (48.5)		
Yes, with related worries	344 (14.1)	193 (14.4)	124 (13.8)	27 (13.8)		
Instrumental ADL, Impaired, n (%)						
	233 (9.6)	102 (7.6)	96 (10.7)	35 (17.9)	χ^2 ^= 22.7	**<.001**
Vision impairment, Impaired, n (%)						
	316 (13.0)	131 (9.8)	145 (16.1)	40 (20.4)	χ^2 ^= 29.4	**<.001**
Hearing impairment, Impaired, n (%)						
	710 (29.2)	321 (24.0)	314 (34.9)	75 (38.3)	χ^2 ^= 39.4	**<.001**
Mobility impairment, Impaired, n (%)						
	780 (32.0)	336 (25.1)	356 (39.5)	88 (44.9)	χ^2 ^= 67.7	**<.001**
Smoking, n (%)						
Never	1,485 (61.0)	775 (57.9)	580 (64.4)	130 (66.3)	χ^2 ^= 13.4	**<.05**
Former	775 (31.8)	453 (33.8)	267 (29.6)	55 (28.1)		
Current	176 (7.2)	111 (8.3)	54 (6.0)	11 (5.6)		
Risky alcohol consumption, n (%)						
	344 (14.1)	205 (15.3)	117 (13.0)	22 (11.2)	χ2 = 3.9	.144
Comorbidity, n (%)						
No diagnosis	351 (14.4)	205 (15.3)	124 (13.8)	22 (11.2)	χ^2 ^= 5.49	.240
1-4 diagnoses	1,888 (77.5)	1,036 (77.4)	693 (76.9)	159 (81.1)		
5+ diagnoses	197 (8.1)	99 (7.3)	84 (9.3)	15 (7.7)		
APOE ϵ4 allele, n (%)						
	498 (20.4)	280 (20.9)	187 (20.8)	31 (15.8)	χ^2 ^= 2.81	.245

^1^Based on the revised version of the international CASMIN educational classification (Brauns and Steinmann, 1999), ADL, activities of daily living; MMSE, Mini Mental Status Examination; SCD, subjective cognitive decline; s.d., standard deviation; sHR, subdistribution hazard ratios. Significant results are displayed in bold fonts.

### 3.2 Incidence of depressive symptoms

Of the total study sample of 2,436 GP patients without significant depressive symptoms at baseline (population at risk), n=543 (22.3%) developed significant depressive symptoms during the study period: the age-specific incidence rate was 33.0 per 1,000 person years (95% CI 28.9-37.8) for individuals aged 75 to 79 years (n=283), 45.5 (95% CI 39.7-52.1) for individuals aged 80 to 84 years (n = 213) and 62.5 (95% CI 45.0-87.4) for individuals aged 85 years and older (n=47), with a significant difference between age groups 75 to 79 and 80 to 84 years, and the age group 75 to 78 and 85+ years.

### 3.3 Age-specific risk factors for incident depressive symptoms

The risk factors for incident depressive symptoms adjusted for mortality were partly similar, partly different in the three age groups (**Table 2**). In the age group 75 to 79 years, female sex (aSHR 1.43, 95% CI 1.01-1.96, p<.05), mobility (aSHR 1.97, 95% CI 1.50-2.58, p<.001) and vision impairment (aSHR 1.64, 95% CI 1.14-2.35, p<.01) as well as SCD without (aSHR 1.43, 95% CI 1.09-1.87, p<.01) or with related worries (aSHR 2.05, 95% CI 1.43-2.94, p<.001) were risk factors for incident depressive symptoms. In the age group 80 to 84 years, the female sex (aSHR 1.69, 95% CI 1.16-2.44, p<.01), being single or divorced compared to married (aSHR 2.00, 95% CI 1.18-3.33, p<.05) mobility (aSHR 1.60, 95% CI 1.20-2.14, p<.01) and hearing impairment (aSHR 1.33, 95% CI 1.02-1.73, p<.05) as well as SCD with related worries (aSHR 2.18, 95% CI 1.44-3.30, p<.001) were risk factors for incident depressive symptoms. In the age group 85+ years, only mobility impairment (aSHR 2.01, 95% CI 1.10-3.70, p<.05) revealed as a risk factor. Unstratified results for risk factors of incident depressive symptoms are reported elsewhere (16).

**Table 2:** Competing risk regression models with mortality and depressive symptoms as competing events (N=2,436).

Characteristics at baseline	75 to 79 years n=1,339 Event: depressive symptoms Competing event: mortality Model I			80 to 84 years n=901 Event: depressive symptoms Competing event: mortality Model II			85+ years n=196 Event: depressive symptoms Competing event: mortality Model III		
	sHR	95% Confidence Interval	p-value	sHR	95% Confidence Interval	p-value	sHR	95% Confidence Interval	p-value
Age									
	1.00	0.91 – 1.10	.955	0.92	0.83 – 1.01	.084	1.08	0.92-1.27	.338
Sex (ref. male)									
	**0.71**	**0.51 – 0.99**	**<.05**	**0.59**	**0.41 – 0.86**	**<.01**	1.24	0.53-2.92	.609
Marital status (ref. single/divorced)									
Married	0.80	0.51 – 1.26	.343	**0.50**	**0.30 – 0.85**	**<.05**	0.42	0.06-3.19	.405
Widowed	0.94	0.62 – 1.41	.754	**0.62**	**0.42 – 0.92**	**<.05**	0.69	0.31-1.56	.375
Living situation (ref. not alone)									
Alone	0.71	0.47 – 1.07	.102	0.84	0.53 – 1.34	.472	1.02	0.25-4.19	.977
Level of education^1^ (ref. low)									
Middle	0.83	0.64 – 1.08	.163	1.12	0.81 – 1.55	.481	0.88	0.49-1.58	.658
High	0.76	0.49 – 1.16	.199	1.42	0.87 – 2.31	.162	0.15	0.01-1.57	.113
MMSE score (per point)									
	0.95	0.89 – 1.02	.159	0.96	0.88 – 1.05	.353	1.01	0.84-1.22	.907
SCD (ref. no SCD)									
Yes, without related worries	**1.43**	**1.09 – 1.87**	**<.01**	1.18	0.87 – 1.59	.292	1.38	0.65-2.96	.402
Yes, with related worries	**2.05**	**1.43 – 2.94**	**<.001**	**2.18**	**1.44 – 3.30**	**<.001**	1.33	0.65-2.73	.439
Instrumental ADL (ref. not impaired) Impaired									
	1.07	0.66 – 1.75	.775	1.35	0.90-2.04	.150	0.66	0.27-1.61	.356
Vision impairment (ref. not impaired) Impaired									
	**1.64**	**1.14 – 2.35**	**<.01**	1.15	0.81 – 1.65	.437	1.01	0.48-2.13	.983
Hearing impairment (ref. not impaired) Impaired									
	0.91	0.69 – 1.22	.538	1.33	1.02-1.73	<.05	0.55	0.26-1.17	.120
Mobility impairment (ref. not impaired) Impaired									
	**1.97**	**1.50 – 2.58**	**<.001**	**1.60**	**1.20 – 2.14**	**<.01**	**2.01**	**1.10-3.70**	**<.05**
Smoking (ref. never)									
Former	1.11	0.85 – 1.47	.438	1.16	0.82 – 1.64	.408	0.89	0.41-1.90	.750
Current	0.89	0.53 – 1.48	.652	1.59	0.93-2.71	0.090	2.84	0.88-9.16	.081
Risky alcohol consumption									
	0.83	0.60 – 1.15	.271	1.08	0.71-1.63	.731	0.51	0.12-2.17	.363
Comorbidity (ref. no diagnosis)									
1-4 diagnoses	1.08	0.77 – 1.50	.655	1.00	0.66 – 1.52	.992	2.77	0.91-8.49	.074
5+ diagnoses	1.11	0.62 – 2.01	.722	0.77	0.40-1.50	.450	0.54	0.05-6.19	.619
APOE ϵ4 allele									
	1.14	0.83 – 1.55	.426	0.98	0.70-1.37	.911	0.91	0.38-2.18	.827

^1^Based on the revised version of the international CASMIN educational classification (Brauns and Steinmann, 1999), ADL, activities of daily living; MMSE, Mini Mental Status Examination; SCD, subjective cognitive decline; s.d., standard deviation; sHR, subdistribution hazard ratios. Significant results are displayed in bold fonts.

## 4 Discussion

The present study aimed to determine age-specific incidence rates as well as age-specific risk factors for incident depressive symptoms adjusting for the competing event of accumulated mortality across the highest age groups of individuals aged 75 to 79 years, 80 to 84 years, and 85+ years.

### 4.1 Incidence of depressive symptoms

The incidence rates were 33, 46 and 63 per 1,000 person-years for the initial age groups 75 to 79, 80 to 84, and 85+ years with a significant difference between the incidence rates of the age group 75 to 79 and 80 to 84 years, and for the oldest-old age group (85+) compared to the age group 75 to 79 years. Skoog et al. (29) reported also higher rates for individuals aged 79 to 85 years of 44 per 1,000 person-years compared to 17 per 1,000 person-years for 70 to 79 year old individuals, but for categorical diagnosis (DSM-III). Only the studies by Harris et al. (30), and Phifer and Murral (31) showed age-group-specific incidences for dimensional measures (by GDS-15 and CES-D, respectively). Harris et al. (30) reported also an increase with increasing age with an incidence proportion of 8.2% for age 75 to 79, 9.8% for age 80 to 84, and 18.3% for age 85+. Phifer and Murral (31) showed an increasing incidence proportion from the age group 75 to 79 (6.6%) to the age group 80 to 84 years (11.8%), but not for the 85+ group (1.3%). The comparison of the results of the present study with the results of earlier studies consistently shows an increase in depressive symptoms with increasing age. It has been shown, that both biological and psychosocial causes contribute to this increase with rising age (32). These include vascular, genetic and general health factors on the one hand, and bereavement, life and social stressors on the other (32).

### 4.2 The risk factors for incident depressive symptoms

We found partly similar and partly differing risk factors for depressive symptoms in the three age groups. Female sex and subjective cognitive decline were only significant risk factors in the two younger age groups (75-79, 80-84 years); these both risk factors do not matter in the oldest age group.

Female sex as a risk factor for depressive symptomatology was reported by many studies for the elderly population [for a summary, see (4, 7)]. In our study, it was only validated for the two younger age groups and while taking cumulated mortality into account, which could be attributed to the higher proportion of women in the oldest age group (85+), and the rather small sample size. Reasons cited for the gender-gap are more dysfunctional coping strategies in elderly women, that woman are much less likely to be married than elderly men, and that they suffer also from poorer health in general than men, all known as risk factors for depression (33). However, it was also pointed out that further research is needed, especially using a model-driven approach (33).

Revising former reviews on risk factors for incident depressive symptoms (7–9), subjective cognitive decline has been shown as the first in the AgeCoDe/AgeQualiDe study (4, 12). In our study, we found subjective cognitive decline as a risk factor for incident depressive symptoms in the two younger age groups (75-79, 80-84 years), but not for the oldest age group. Slot et al. (34) found that subjective cognitive decline could be the first notable manifestation in the preclinical stage of Alzheimer’s disease (AD), while Jessen et al. (17) indicated that subthreshold depressive symptoms may also be manifestations of preclinical AD. Kleineidam et al. (35) reported that subjective cognitive decline precedes depressive symptoms in the development of dementia.

Plassman et al. (36) estimated the mean age of onset of a dementia diagnosis to be 83.7 years. Conclusively, one could assume that these two age groups are the groups being in the highest risk of a preclinical dementia stage.

The marital status of being single or divorced was only a significant risk factor in the age group 80 to 84 years, but not in the younger and oldest age group. Previous reviews showed rather heterogeneous or insignificant results for marital status (4, 9) that support our findings of significance only for the age group 80 to 84 years. Sociodemographic information of our sample shows an increasing number of individuals being single or divorced, as well as widowed, and a decreasing number of married individuals across the considered age range, and also an increasing number of individuals living alone. The age between 80 and 84 years seems to be a vulnerable age of loss of the partner or spouse, since the average life expectancy was reported to be 17.9 years for 65-year-old men and 21.1 years for 65-year-old women in 2018 (37). Since we know that more often women survive their spouses and with a view to the proportion of women in our sample, this finding could support our assumption of the higher vulnerability of women in the age group 80 to 84 years.

Mobility impairment was a significant risk factor for the development of depressive symptoms in the three age groups. Likewise, Chou (38) and Weyerer et al. (12) showed this risk factor for depressive symptoms for samples of 65+ and 75+ years. Vision impairment was found to be only a risk factor for the age group 75 to 79 years and hearing impairment only for the age group 80 to 84 years. Vision and mobility impairment were also found to be a risk factor for depressive symptomatology in most of the studies included in the recently published systematic review by Maier et al. (4), while hearing impairment could not be confirmed by the findings of Maier et al. (4). Loss of mobility has been described as a downward curve with a steeper decline in later life, which occurs when the ability to compensate for the cumulative effect of impairments is exhausted (39). This may have occurred in many of the study participants, since the proportion of people with mobility impairment increased significantly in all age groups of the study sample.

### 4.3 Limitations

First, in studies with voluntary participation, participation bias can never be ruled out. Although the selection of risk factors was hypothesis-driven, based on the findings and shortcomings of the studies of risk factors of incident depression in current reviews (4, 7), we might have ignored other potential factors. Furthermore, exclusion criteria for insufficient language skills, blindness, deafness, living in a nursing home, as well as lack of ability to provide informed consent, may have affected the findings of incident depression. Furthermore, depressive symptoms were not assessed using the DSM-V criteria. However, GDS is a commonly used instrument in epidemiological studies, and depressive symptoms were evaluated by trained physician’s and psychologists.

## 5 Conclusions

Depressive symptoms in the highest age are common and lead to a high individual, familial, and societal burden. Our findings contribute significantly to the available knowledge about risk factors for depression in the oldest population. So far, studies that investigated risk factors did not take into account competing event mortality, thus neglecting death as a frequently occurring event in that age group. Furthermore, only a few studies focused on the highest age groups. With our findings, both gaps were closed. Addressing these aspects in further prospective studies conducted in the highest age groups may provide additional information on the mental state of this rapidly growing age group. In fact, the majority of identified risk factors of late life depression are modifiable and the finding of different risk factors in the highest age groups opens the possibility of specifically tailored prevention concepts.

## Data availability statement

Aggregated data are provided in the article tables. The raw datasets presented in this article are not readily available due to ethical restrictions and patient confidentiality, but are available upon request from the Working Group Medical Statistics and IT-Infrastructure. Requests to access the datasets should be directed to BW, wiese.birgitt@mh-hannover.de.

## Ethics statement

The studies involving humans were approved by the Ethics Committees of all participating study centers: − Ethics Commission of the Medical Association Hamburg (reference number: MC-390/13) − Ethics Committee of the Medical Faculty of the Rheinische Friedrich-Wilhelms-University of Bonn (reference number: 369/13) − Medical Ethics Commission II of the Medical Faculty Mannheim/Heidelberg University (reference number: 2013-662 N-MA) − Ethics committee at the Faculty of Medicine of the University of Leipzig (reference number: 309/2007; 333-13-18112013) − Ethical Committee of the Medical Faculty of the Heinrich-Heine-University Düsseldorf (reference number: 2999) − Ethics committee of the Faculty of Medicine of the Technical University of Munich (713/02 E) and comply with the ethical standards of the Declaration of Helsinki. The studies were conducted in accordance with the local legislation and institutional requirements. The participants provided their written informed consent to participate in this study.

## Author contributions

MeL: Formal analysis, Investigation, Writing – original draft, Visualization. AP: Formal analysis, Writing – original draft, Writing – review & editing. MaL: Investigation, Writing – review & editing. TM: Investigation, Writing – review & editing. CB: Writing – review & editing. AH: Writing – review & editing. KH: Investigation, Writing – review & editing. LK: Writing – review & editing, Investigation. SW: Writing – review & editing. JW: Investigation, Writing – review & editing. MP: Investigation, Writing – review & editing. DW: Investigation, Writing – review & editing. EM: Investigation, Writing – review & editing. BW: Data curation, Writing – review & editing. AO: Data curation, Writing – review & editing. MW: Conceptualization, Funding acquisition, Project administration, Writing – review & editing. WM: Conceptualization, Funding acquisition, Project administration, Writing – review & editing. MS: Conceptualization, Funding acquisition, Project administration, Writing – review & editing. H-HK: Conceptualization, Funding acquisition, Project administration, Writing – review & editing. SR-H: Conceptualization, Formal analysis, Funding acquisition, Project administration, Writing – original draft, Writing – review & editing.
