# Supplementary material for: Age-specific risk factors of depression among the oldest-old - evidence from the multicenter AgeCoDe-AgeQualiDe study
Source: Front Psychiatry. 2024 Jun 11;15:1367225. doi: 10.3389/fpsyt.2024.1367225 (PMC11196990; doi:10.3389/fpsyt.2024.1367225)
Supplement: Supplementary file 1 [file Table_1.docx]

**Supplementary file 1: Ethics commitee names, approval codes and dates**

| **Center** | **Name of Ethics commitee** | **Approval code/date** | | |
| --- | --- | --- | --- | --- |
|  |  | **Funding 1**  **(Baseline –FUII)** | **Funding 2**  **(FUIII – FUVI)** | **Funding 3**  **(FUVII-FUIX)** |
| Hamburg | Ethics Commission of the Medical Association Hamburg | OB / 08 / 02 | 2817/2007 | MC-390/13 |
| Bonn | Ethics Committee of the Medical Faculty of the Rheinische Friedrich-Wilhelms-University of Bonn | 050/02  und 174/02 für E 3.2 | 258/07 | 369/13 |
| Mannheim | Medical Ethics Commission II of the Medical Faculty Mannheim/Heidelberg University | 0226.4 /2002 | 2007-253E-MA | 2013-662N-MA |
| Leipzig | Ethics committee at the Faculty of Medicine of the University of Leipzig | 143/2002 | 309/2007 | 309/2007;  AZ: 333-13-18112013 |
| Düsseldorf | Ethical Committee of the Medical Faculty of the Heinrich-Heine-University Düsseldorf | 2079/2002 | 2999/2008 | 2999 |
| München | Ethics committee of the Faculty of Medicine of the Technical University of Munich | 713/02 | 713/02 E | 713/02 E |
